# Bronchial epithelial cell-derived extracellular vesicle analysis using conventional, imaging, and nanoscale flow cytometry technologies

**DOI:** 10.1038/s41598-026-41848-x

**Published:** 2026-02-26

**Authors:** Georgina Hopkins, William Browne, Davis Tucis, Stella Cochrane, Victoria James, David Onion, Lucy C. Fairclough

**Affiliations:** 1 School of Life Sciences, The University of Nottingham, Nottingham, NG7 2UH UK; 2 SERS, Unilever, Colworth Science Park, Sharnbrook, Bedfordshire, MK44 1LQ UK; 3 School of Veterinary Medicine and Science, The University of Nottingham, Nottingham, NG7 2UH UK

**Keywords:** Extracellular vesicles, Tetraspanins, Flow cytometry, NTA, Epithelial cells, Biological techniques, Biotechnology, Cell biology

## Abstract

Bronchial epithelial cell-derived extracellular vesicles (EVs) are central to airway immune responses to inhaled particulate stimuli, as well as regulating respiratory diseases. Through a complex bioactive cargo, EVs can influence inflammatory signalling in the epithelium. Typically, a variety of technologies are used to analyse EVs derived from biofluids, such as nanoparticle tracking analysis, western blotting, and transmission electron microscopy. But recent advances in flow cytometers (FCs) potentially provide a single technology that can rapidly enumerate, size and phenotype epithelial cell-derived EVs, without the absolute need for purification. With multiple FCs available, this study aimed to describe methods and discuss considerations for analysing epithelial cell-derived EV on different FCs. Thus, supernatants containing EVs from primary human bronchial epithelial cells were stained with calcein-AM, in combination with anti-fluorescently conjugated tetraspanin antibodies, before analysing on a CytoFLEX S, ImageStream X MKII, and CytoFLEX nano. NIST traceable polystyrene particle size standards or synthetic EV size standards were used for EV size calibration, and antibody capture microspheres were used to measure the limit of detection for tetraspanin antibodies. We demonstrated that epithelial cell-derived EVs can be sized, enumerated, and phenotyped using all tested FC technologies, with varying sizing sensitivities and considerations for each FC. These findings provide a guidance for selecting suitable FC technologies for EV characterisation and highlight their potential to dissect epithelial EV heterogeneity in the context of airway immune responses and inflammation.

**Supplementary Information:**

The online version contains supplementary material available at 10.1038/s41598-026-41848-x.

## Introduction

Extracellular vesicles (EVs) are lipid bound vesicles released by all cells into most bodily fluids, such as blood, plasma and urine^1^. EVs play a role in cellular communication, with increasing evidence for their role as biomarkers and therapeutic agents in various diseases, such as cancer^2^. Specifically, EVs secreted by human bronchial epithelial cells (HBEC-EVs) have been demonstrated in immune responses to inhaled particulates and in respiratory diseases, including regulating the development of allergic sensitisation^3,4^, reducing fungal infection in the lungs^5^, promoting airway remodelling in chronic obstructive pulmonary disease (COPD)^6^, and inhibiting lung fibrosis^7^. Hence, HBEC-EVs play a key role in regulating the lung microenvironment and have potential as therapeutics or as biomarkers for respiratory diseases.

However, as EVs are small (30–1000 nm in diameter) ^8,9^, heterogeneous, and have a low epitope copy number, they have proven difficult to measure. Conventionally, EVs are isolated by ultracentrifugation, density-gradient, or size exclusion chromatography (SEC), followed by nanoparticle tracking analysis (NTA) to size and quantify, or transmission electron microscopy (TEM) to size and image. These methods are time consuming, lack throughput, and lack the ability to confirm phenotype of vesicle being analysed. To enable HBEC-EV analysis in translational studies or a clinical setting, high-throughput multi-parametric methods, such as flow cytometry (FC), would be highly advantageous.

Flow cytometry is a powerful tool originally designed for single-cell analysis. Using light scattering and fluorescence signals from single particles, the development of advanced flow cytometers (FCs) with high sensitivity, such as nanoscale FCs, has increased their application in EV analysis, now being one of the most common methods^10^. Conventional FCs are designed for larger particle analysis; thus, they tend to have lower sizing resolution and high instrument noise, which can complicate the detection of EVs. Conversely, some imaging FCs combine flow cytometry with high-resolution microscopy; with a longer exposure time and higher signal to noise ratio due to CCD camera detection, leading to higher sensitivity and lower electronic noise in comparison to most conventional FCs. However, the throughput is lower than conventional FCs as sample processing is slower. More recently, commercially available, nanoscale FCs have been developed, which are specialised in detecting and phenotyping particles as small as 35–40 nm^11,12^. However, as nanoscale FCs are not yet widely adopted it is important to understand the relative merits of all 3 types of FCs.

Fluorescent dyes can provide a stable and strong signal for EV detection by conventional, imaging, and nanoscale flow cytometry. Calcein acetoxymethyl (AM) consists of fluorescent calcein combined with acetoxymethyl group, and works by penetrating the lipid bilayer before being cleaved by cytosolic esterases, resulting in a water-soluble fluorophore when the EV is intact^13,14^. Calcein-AM could be advantageous compared to other EV dyes, such as lipophilic membrane dyes, which have been shown to produce non-specific fluorescent particles that are indistinguishable from labelled EVs, confounding experimental results^15–17^. Utilisation of EV dyes in conjunction with fluorescent antibodies allows the quantification of multiple EV biomarkers by flow cytometry, including the common EV antigens; tetraspanins CD9, CD63, and CD81. As opposed to other methods for tetraspanin analysis, such as western blotting, which is only semi-quantitative.

A framework designed to complement MISEV guidelines^1^, MIFlowCyt-EV, has supported the standardised reporting of sample staining, EV detection, measurement, and experimental design in FC data^18^. However, with increasing FC technologies available to study EVs, there is a lack of clarity over the suitability of each technology for EV research. Thus, building upon previous methods for flow cytometry analysis of EVs^19–26^, we further examined methods for EV quantification, sizing, and protein characterisation, without the need for prior EV isolation, which is required for other EV technologies, such as TEM and NTA, to study HBEC-EVs. In contrast to previous FC comparison studies^7^, we utilised a range of flow cytometry technologies: conventional, imaging, and nanoscale FCs, to describe specific considerations for EV analysis by each FC, to ensure that any data generated is robust and limitations are acknowledged. This study aims to provide clarity on what methods and instruments are best suited to study HBEC-EVs, which has not been shown previously.

## Materials and methods

### EVs derived from primary human bronchial epithelial cells

MucilAir™ were purchased from Epithelix Sárl; a fully differentiated bronchial epithelial model using primary human cells from healthy donors. A quality control sheet was provided by the manufacturer detailing the batch TEER measurement, mycoplasma testing result, cilia beating frequency, mucus presence, and timings from seeding to fully-differentiated ALI. The samples arrived in the format of 24-well transwell inserts which were then placed in a sterile 24-well plate with phenol red-free and serum-free MucilAir™ culture medium (Epithelix Sárl) in a humidified incubator (37 °C; 5% CO_2_), replacing with MucilAir™ media every 2–3 days. The MucilAir™ culture medium containing EVs was aspirated and used for subsequent analysis.

### Size exclusion chromatography

Before Transmission Electron Microscopy (TEM), super-resolution microscopy, Nanoparticle Tracking Analysis (NTA), and CytoFLEX nano analysis, cell supernatants were removed from wells and centrifuged at 300 g for 8 min to remove any contaminating cells. EVs were then isolated by SEC using qEV original 35 columns with an automated fraction collector (Izon Science). Fractions 1–7 of 500 µL were collected, pooled, and concentrated by centrifuging at 4000 g for 10 min using 10 kDa amicon columns (Merck). The result is ~ 250 µL of EVs in solution, ranging from 35 nm to 350 nm in size.

### Transmission electron microscopy

SEC isolated EVs were placed on carbon-coated 300 Cu mesh TEM grids (company) for 15 min and then fixed with 2% paraformaldehyde for 10 min. The grids were washed 5 times with deionised water before incubating with 4% Uranyl Acetate for 10 min. Excess stain was removed before allowing to dry completely, and then grids were imaged using a Tecnai BioTwin TEM, operating at 80 kV, and images were recorded on a Gatan Orius camera.

### Super-resolution microscopy

SEC isolated EVs were stained with tetraspanin antibodies tagged with blinking fluorophores (EV profiler Kit, EV-MAN-1.0, Oxford Nanoimaging) (CD81-647, CD63-561 and CD9-488) for 60 min at 4 °C. Pre-coated slide with capture antibodies was provided by the company, which was then blocked with 5% BSA. The labelled EVs were then placed on the slide and incubated for 60 min at room temperature. To ensure higher clarity of data acquired, the slides were fixed with 4% formaldehyde for 10 min. Setting up the ONI supermicroscope was done according to the manufacturer’s instructions. Briefly, focus, laser power, exposure and frame rate were adjusted based on the established protocols set by the manufacturer to utilise the single particle tracking (SPT). The data acquired was analysed using the manufacturer’s software CODI.

### Nanoparticle tracking analysis

SEC isolated EVs were diluted 1 in 1000 in 0.22 µM filtered PBS and inserted into an LM10/14 Nanosight instrument (Nanosight, Malvern Panalytical, UK) to determine particle size distribution and quantification. Prior to analysis, a 1:10 dilution of 100 nm carboxylated polystyrene (IZON) and a 1:1000 dilution of 200 nm polystyrene (Malvern Panalytical) nanoparticles were used to calibrate the sensitivity of the instrument. Automatic settings were applied for the minimum expected particle size, minimum track length, and blur settings, with 11 positions. Sensitivity was set at 78, gain at 26.88, and temperature set at 21 °C. National Institute of Standards and Technology (NIST) beads were run at a sensitivity of 65, in technical repeats of 11. Data processing and analysis of particle size distribution were performed using NTA Software 3.3 Dev build 3.3.301 (Malvern Panalytical).

### Flow cytometer acquisition settings

A conventional flow cytometer (CytoFLEX S, Beckman Coulter), nano flow cytometer (CytoFLEX nano, Beckman Coulter), and imaging flow cytometer (ImageStream X MKII, Cytek Biosciences) were tested for measuring EVs. The instrument acquisition settings for each cytometer are listed below. Configuration and gain settings for each FC are provided in the supplementary methods.

#### CytoFLEX S:

Violet side scatter and high event rate detection were enabled and a threshold of 40,000 violet side scatter-height was set. A flow rate of 10 µL/min was set and 20 µL of sample acquired.

#### CytoFLEX nano:

Using CytExpert nano software, the most sensitive channel, VSSC1, was selected for the threshold and set to 265. The sample flow rate was set to 1 µL/min and 2 µL of sample was recorded.

#### ImageStream X Mk II:

High Gain was turned on and magnification was set to 60x. The fluidics were set to low speed with high sensitivity, resulting in a flow speed of 44 mm/sec and ~ 1 µL of sample was recorded.

### Sizing of EVs by flow cytometry

National Institute of Standards and Technology (NIST) certified polystyrene size standards (60 nm, 80 nm, 101 nm, 125 nm, and 151 nm, (ThermoFisher 3060 A, 3080 A, 3125 A, 3100 A, and 3150 A)), were utilised on ImageStream X Mk II, CytoFLEX S, and NTA. The beads were diluted in 0.22 μm sterile-filtered PBS accordingly for each machine; 1 × 10^− 4^−1 × 10^− 5^ for ImageStream X MkII, − 1 × 10^− 5^ for CytoFLEX S, and − 1 × 10^− 6^ for NTA. For the CytoFLEX nano, NIST certified polystyrene size standards (80 nm, 101 nm, 200 nm, 300 nm, and 450 nm, (Thermo Fisher 3080 A, 30100 A, 3200 A, 3300 A, and 3450 A)) were used and diluted at 2.5 × 10^− 7^/mL. Light scattering signals of NIST bead populations run on the CytoFLEX S and CytoFLEX nano were then fitted with Mie theory using FCMPASS software’s default core-shell model^27,28^. The refractive index of each bead (provided by the manufacturer) was inputted into the software. Effective scattering cross sections of the calibration beads were calculated by integrating the amplitude scattering matrix elements over 576 collection angles. Data and theory were log10-transformed to scale the data onto the theory using a least-square-fit.

For the ImageStream X MkII, the vFC™ EV analysis assay kit (Cellarcus Biosciences) was used to determine EV size, and performed according to the manufacturer’s instructions. Briefly, the instrument was first calibrated using vCal™ nanoRainbow beads and the manufacturer’s provided IDEAS layout. 5 µL of a synthetic vesicle size standard, Lipo100™, or 5 µL primary epithelial cell supernatant, were then stained with a membrane stain, vFRed™, at 1X concentration in vFC™ staining buffer, in a total volume of 50 µL for 1 h at room temperature, in the dark. Samples were then diluted 1:30 in staining buffer before running on the ImageStream X MkII. Files were imported into IDEAS software and exported as FCS files, before analysing in FCS Express (DeNovo software) using the manufacturer’s provided layouts. Using the Lip100™ size standard, with a size range of 80–140 nm as determined by NTA, vFRed-positive events were selected and calibrated using FCS Express transformations tool and inputting a surface area equation generated by the manufacturer’s layout. This size calibration was then applied to samples from primary epithelial cells to size the EVs.

### Limit of detection

Anti-Human and Anti-Mouse IgG Quantum™ Simply Cellular fluorescent standardisation beads were assessed on ImageStream X MkII and CytoFLEX according to the manufacturer’s instructions (Bangs Laboratories, Fishers, IN, USA). LoDs were determined using product-specific templates provided by the manufacturer. All instrument lasers were set to max power for LoD analysis.

### Staining of EVs for flow cytometry

Supernatants from primary bronchial epithelial cells were removed from wells and centrifuged at 300 g for 8 min to remove any contaminating cells. EV-containing supernatants or isolated EVs, in a volume of 50 µL, were then stained with a final concentration of 10 µM calcein-AM (Biolegend) and vortexed for 1 min, before incubating for 1 h at 37 °C, in the dark. When identifying tetraspanin expression, a final concentration of 2.5 µg/mL of anti-tetraspanin monoclonal antibodies for CD9 APC, CD63 PE, and CD81 PE-Vio615 were added at the same time as calcein-AM. The samples were then diluted in 0.22 µM filtered PBS at ratios of 1:100 for the CytoFLEX S, or 1:7 for the ImageStream X MkII. Tetraspanin gates were set using isotype control for each antibody. Controls for EV staining included an unstained sample, PBS buffer with calcein, culture medium with calcein, and 10% of Sodium dodecyl sulphate (SDS) was added to a calcein-stained sample for 30 min at RT before analysis.

For the CytoFLEX nano, supernatants from primary bronchial epithelial cells were removed from wells and centrifuged at 300 g for 8 min to remove any contaminating cells, but also subsequently isolated by SEC. Isolated EVs, in a volume of 50 µL, were then stained with a final concentration of 10 µM calcein-AM (Biolegend) and vortexed for 1 min, before incubating for 1 h at 37 °C, in the dark. When identifying tetraspanin expression, a final concentration of 2.5 µg/mL of anti-tetraspanin monoclonal antibodies for CD9 Vioblue, CD63 PE, and CD81 APC (Milteneyi Biotec) were added at the same time as calcein-AM. The samples were then diluted 1:20 in 0.22 µM filtered PBS and run on the CytoFLEX nano.

Compensation was performed on all machines using single-stained EV samples to ensure accurate correction at the low fluorescence intensities characteristic of EVs.

### Data and statistical analysis

Flow cytometry data collected using CytoFLEX S and CytoFLEX nano machines were analysed with Kaluza Analysis 2.1 software. Data collected using the ImageStream X MkII was analysed using IDEAS 6.2 software and subsequently FCS Express 7.24 (DeNovo) for sizing. results were then imported into Prism software, version 10.4.1 (GraphPad), for statistical analysis.

## Results

### Microscopy technologies for EV characterisation

**Fig. 1:**
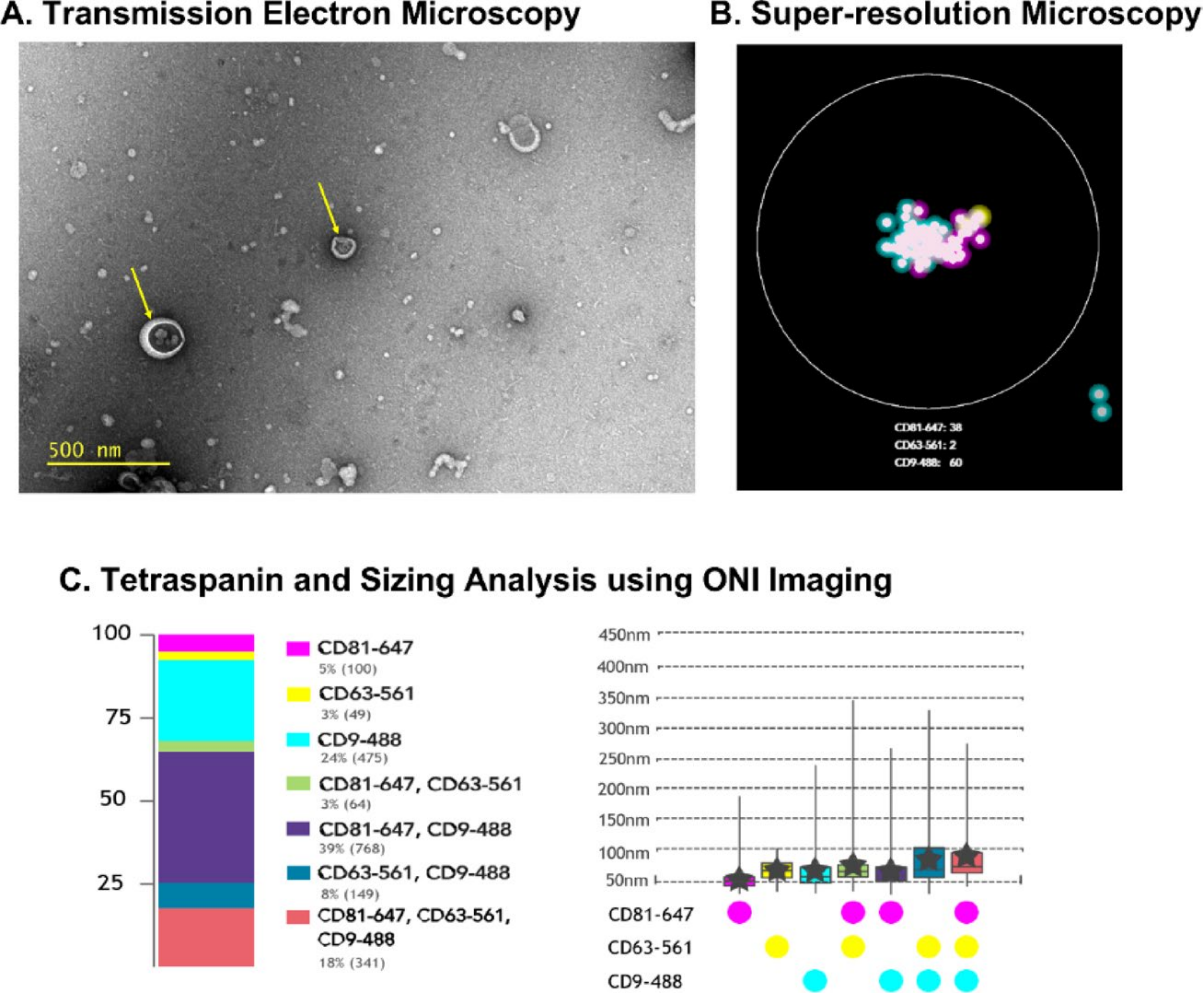
Microscopy EV Technologies. EVs derived from primary human epithelial cells were imaged by TEM **(A)** and subsequently imaged by super-resolution microscopy **(B)** to determine tetraspanin expression and size **(C)**.

Before evaluating flow cytometry techniques for HBEC-EV detection and characterisation, two microscopy technologies were used to confirm (i) the presence of EVs in HBEC supernatants and (ii) that EVs were expressing tetraspanins, in agreement with MISEV guidelines^1^. Firstly, using primary epithelial EVs isolated by SEC, TEM confirmed the presence of small EVs (< 200 nm) in the cell supernatant, showing spherical, cup-shaped, membrane enclosed particles consistent with the morphology of EVs (Fig. 1A). Super-resolution microscopy (ONI), which captures EVs using microfluidic chips and detects tetraspanins using fluorescent reagents^29^, revealed clusters of tetraspanins present on single EVs (Fig. 1B). The tetraspanin expression was then quantified across the sample, estimating the size of each EV using tracking-based methods, showing EVs were all under 350 nm is size (Fig. 1C). Despite the high sensitivity of super-resolution microscopy, the technology is not high-throughput and so there is a need to explore other technologies that can accurately quantify, size and phenotype EVs, such as flow cytometry.

### Sizing of extracellular vesicles

NTA is currently the most commonly used technology for quantifying and sizing EVs. However, advances in the flow cytometry field have enabled the sizing of EVs, in addition to their quantification and phenotyping, allowing users to get multiple readouts with one technology. To determine the size of EVs by flow cytometry, nanosphere particles of specific sizes can be used to generate a curve by Mie Theory. Here, we utilised polystyrene nanosphere particles of sizes 40–151 nm and interpolated the sizes on a conventional FC (CytoFLEX S), and nano FC (CytoFLEX nano), using NTA as a comparison. The EVs detected in the following experiments were derived from human primary bronchial epithelial cell supernatants, without the need for isolation.

**Fig. 2:**
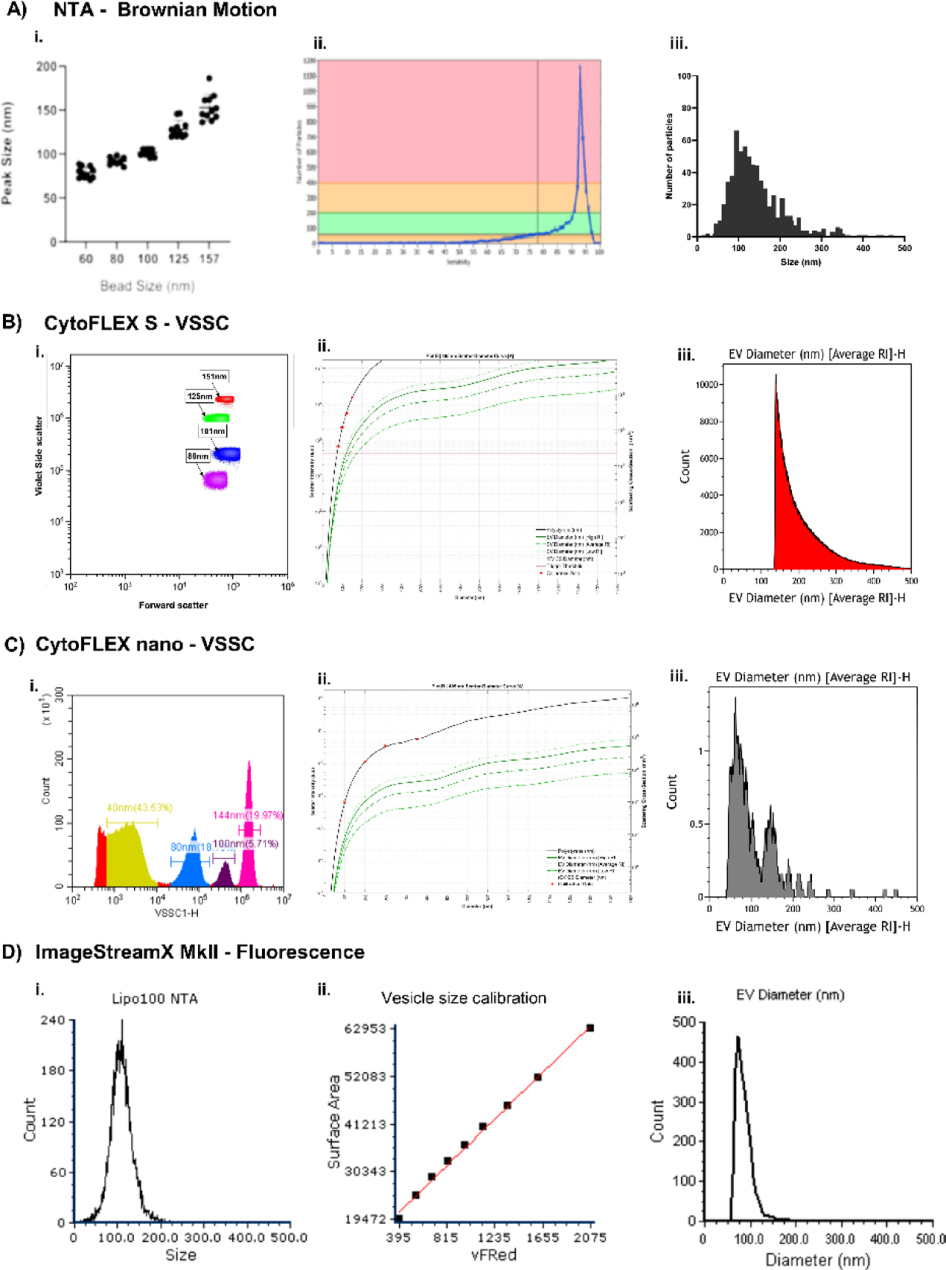
EV sizing methods using **(A)** NTA, and FC instruments **(B)** CytoFLEX S, **(C)** CytoFLEX nano, and **(D)** ImageStream X MkII. (Ai) NIST beads were run on NTA and (Aii) the sensitivity of the machine was set at 78. (Aiii) Using Brownian motion, NTA sized EVs derived from primary human epithelial cells. (Bi) NIST beads were also run on the CytoFLEX S FC using violet side scatter, to then be used for (Bii) Mie theory conversion, (Biii) allowing EVs from epithelial cells to be sized. Similarly, (Ci) NIST beads were run on the CytoFLEX nano FC and used for (Cii) Mie theory conversion, (Ciii) allowing EVs from epithelial cells to be sized. (Di) The imaging FC, ImageStream X MkII, employed a vesicle size standard confirmed by NTA to then be used for (Dii) calibration with a fluorescent membrane stain. (Diii) The calibration file could then be applied to primary epithelial EV samples stained with the VFRed dye for sizing.

NTA uses Brownian motion to size particles, and it is important to set the right sensitivity for particle detection. After inserting any sample into the machine, sensitivity can first be calculated by looking at the sample in both the analogue and digital view; if it is too low, very few particles are present, but if it is too high, the emitted light distorts the size of the particles, causing them to merge. Thus, sensitivity must be set so that most particles are visualised but are still round and not merged. Sensitivity can be further clarified by the use of the ‘detect particle sensitivity’ function. The size of NIST beads was first examined, with the sensitivity set to 65 using the digital view to optimise the visualisation of the beads as individual beads and minimising the background across all the bead sizes. Figure 2Ai shows that NTA detects NIST beads in peak sizes similar to the actual bead size. For HBEC-EVs, sensitivity was set to 78 using the analogue and digital view (Fig. 2Aii). Once this sensitivity value is decided, this should be kept the same for all biological samples within an experiment. Other settings applied were a temperature of 21 °C, as heat can increase the movement of particles and change the size readouts. The NTA detected EVs as low as 20 nm within the primary epithelial EV sample (Fig. 2Aiii).

The conventional FC, CytoFLEX S, was then evaluated for EV sizing. Figure 2Bi demonstrates the 4 NIST beads detected by the machine (80–151 nm), with the smaller 60 nm bead not distinguishable from the background of the machine. The 4 bead sizes were then input into FCMPASS software for Mie Theory conversion, which considers the refractive index of the NIST beads and light scattering abilities (Fig. 2Bii). The 80 nm beads equated to 126 nm in EV size after Mie Theory interpolation. Thus, all EVs below this size are lost in ‘noise’ of the machine, when triggering using the VSSC setup utilised here. Thus, when subsequently sizing primary epithelial-derived EVs using the NIST beads, the smallest EVs detected were 126 nm, ranging up to ~ 500 nm (Fig. 2Biii). It must be noted that to achieve this level of sensitivity on the CytoFLEX S, the samples must be run using the violet side scatter (VSSC) instead of regular side scatter to improve resolution.

The CytoFLEX nano sizes EVs in the same manner as the CytoFLEX S, but as the nano FC is more sensitive due to its improved optics, higher sensitivity detectors, optimised scatter detection, and improved fluidics system, it can detect NIST beads as small as 40 nm (Fig. 2Ci), which can then be input into FCMPASS software for Mie Theory conversion (Fig. 2Cii). EVs as small as 40 nm were detected using this FC (Fig. 2Ciii). Although, for EVs to be run on the CytoFLEX nano, it was recommended to isolate EVs before analysis. Thus, the representative epithelial sample was isolated by automated SEC.

At the time of publication, the imaging FC, ImageStream X MkII, was not set up to use FCMPASS software for Mie Theory conversion for sizing EVs. As an alternative, a commercially available kit was used, which provides size estimation by quantitative measurement of lipid dye incorporation in comparison to a well characterised synthetic lipid size standard. The synthetic size standard has a size range of 80–140 nm as determined by NTA (Fig. 2Di), which can be stained with the membrane dye and calibrated using FCS Express transformations tool and inputting a surface area equation generated by the manufacturer’s layout (Fig. 2Dii). The intensity of vFRed™ in stained samples can then be used to interpolate the size of the EV samples. The primary epithelial EV sample demonstrates EVs as small as 55 nm were present (Fig. 2Diii). This method was also tested on the CytoFLEX S to see if sizing by fluorescence could enable more sensitive detection of EVs, compared to the 126 nm sensitivity when using NIST beads with VSSC. However, the CytoFLEX S was not efficient at detecting the Lipo100™ standard, thus the sizing could not be directly calibrated using fluorescence. This method was not tested on the CytoFLEX nano due to limited instrument access.

### Enumeration of extracellular vesicles

Many existing studies use lipid-membrane dyes to stain EVs for flow cytometry. However, these dyes can stain EV fragments and thus can give false measurements^15–17^. Alternatively, intact EVs can be identified by staining with Calcein-AM, a membrane-permeable dye which is hydrolysed by the enzyme esterase to a polar green-fluorescent product (calcein).

**Fig. 3:**
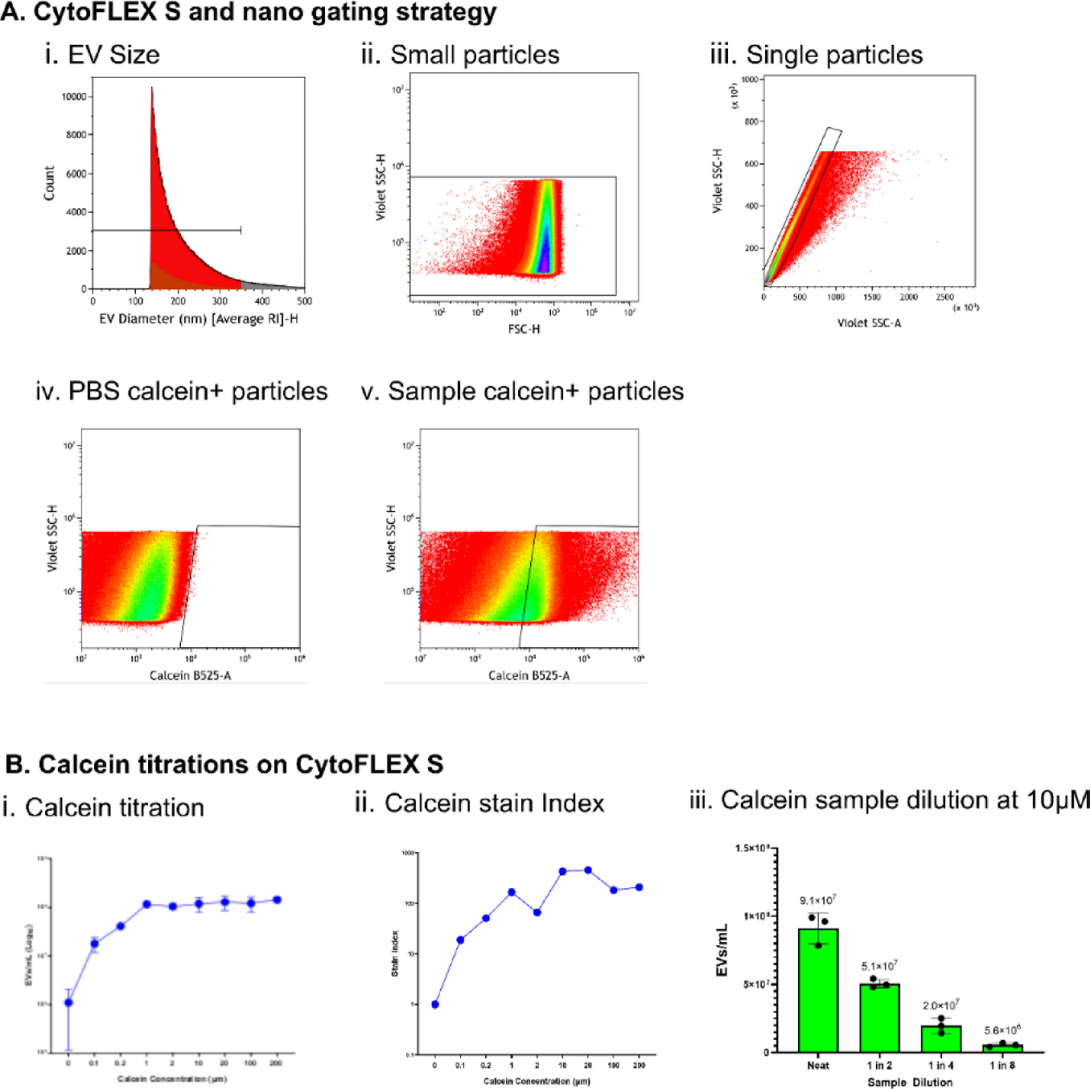
Calcein Titrations. **(A)** EV gating strategy on CytoFLEX S and nano. **(i)** Data is first run through FCMPASS to obtain the size distribution of EVs. EVs less than 300 nm were then gated. **(B)** (ii) Sterile-filtered PBS and MucilAir supernatant were stained with increasing concentrations of calcein-AM and run on CytoFLEX S. (iii) Serial dilutions of primary epithelial cell supernatant were performed and stained with 10 µM of calcein to determine any swarming at high concentrations of EVs, before analysing. (*n* = 3).

Using the conventional FC, CytoFLEX S, EVs can be measured using calcein-AM through the gating strategy presented in Fig. 3A. Using sizing data from FCMPASS (Fig. 3Ai), a 300 nm size gate can be applied to a VSSC-H/FSC-H plot to select only small particles (Fig. 3Aii). Single particles are then selected by plotting VSSC-H/VSSC-A (Fig. 3Aiii). Calcein-AM has been shown to autofluoresce, thus, a PBS + calcein control was used to create a gate for calcein-positive events (Fig. 3Aiv). Finally, using a primary human bronchial epithelial supernatant sample stained with calcein-AM, the gate can be applied to measure the number of EVs present in the sample (Fig. 3Av). The CytoFLEX nano can be used in the same manner, with the same gating strategy.

Calcein-AM is often utilised for staining cells and not EVs, thus the optimal concentration for staining EVs has not been fully identified. Building upon a limited range of calcein titrations conducted in previous work^14^, here, supernatant from primary human epithelial cells was isolated and stained for 8 calcein concentrations between 0.1 µM and 200 µM. The samples were subsequently run on CytoFLEX S to determine the optimal concentration. The results are presented in Fig. 3B with readings from PBS+calcein samples at each titration subtracted to remove any autofluorescence. The results in Fig. 3Bi indicate calcein-positive EV detection begins to plateau at a concentration of 1 µM of calcein, but the stain index of calcein was optimal at 10 µM of calcein (Fig. 3Bii). Thus, a 10 µM concentration was chosen to stain EVs in subsequent experiments. Serial sample dilutions and staining with 10 µM of calcein demonstrated a linear trend in decreasing EV numbers with increasing sample dilution (Fig. 3Biii). This indicates the calcein-AM is efficiently staining all EV sample concentrations tested.

**Fig. 4:**
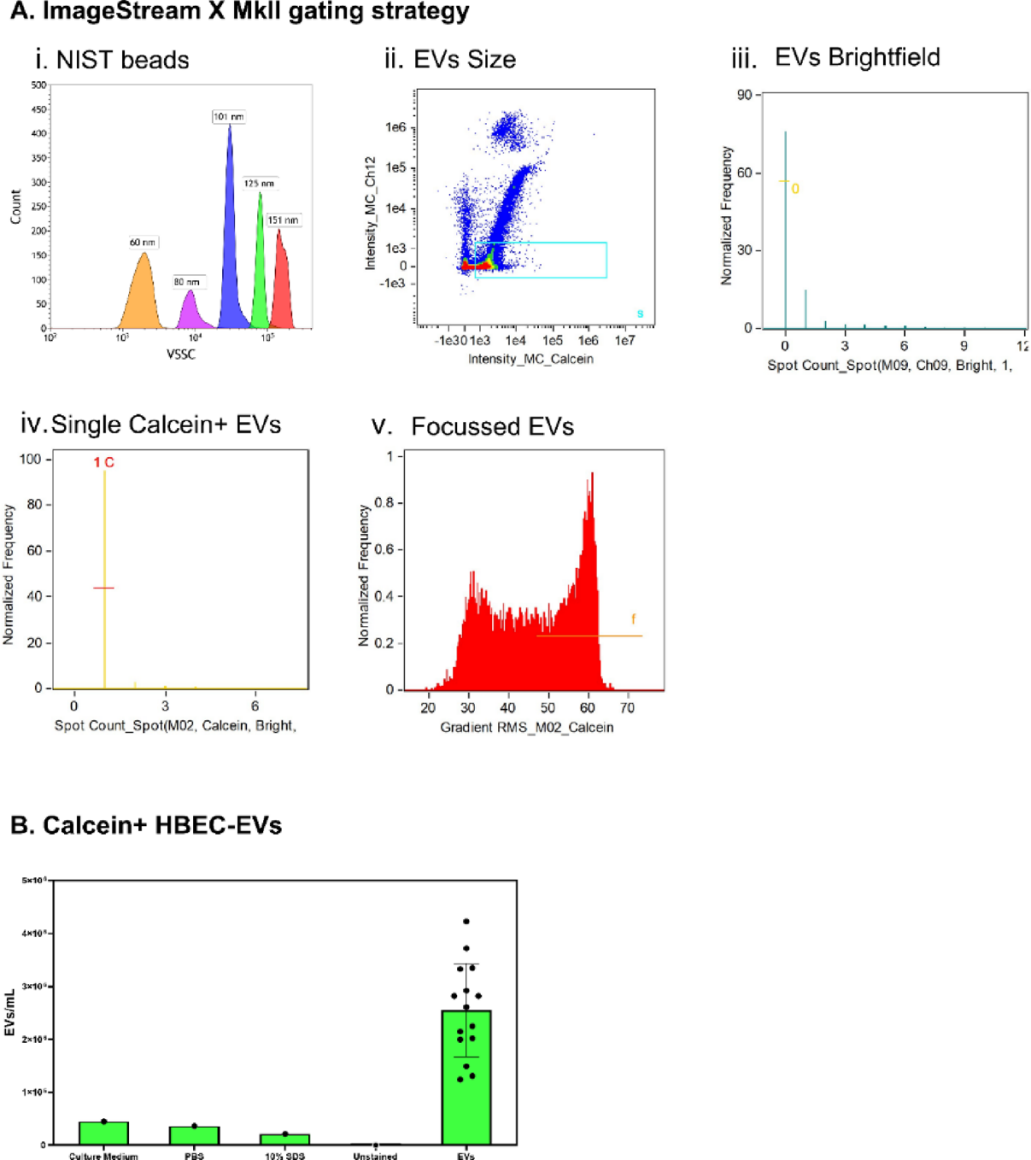
ImageStream X MkII gating strategy. **(A)** Gating strategy on ImagestreamX MkII. (i) NIST beads of 60–151 nm were used to then (ii) create a gate (blue) to select everything below the 151 nm beads position against calcein intensity. (iii) To ensure no large events were selected, a spot count in brightfield channel was applied. (iv) Only calcein-positive events were selected. (v) Only in-focus events were selected **(B)** Calein+ HBEC-EVs with MISEV-recommended controls (*n* = 15).

The imaging FC, Imagestream X MkII, requires a different gating strategy for EV quantification due to the use of cameras. In order to accurately distinguish EVs from the background, a series of gates were used in IDEAS software. First, the EVs were gated on their size by utilizing a series of nanobeads ranging from 60 to 151 nm in size (Fig. 4i and ii). However, during analysis on the IDEAS software, an extra gate was applied, where artefacts were removed from the gated data using a brightfield spot count feature and any events with a brightfield image were excluded (Fig. 4iii). In order to only examine individual EVs, as mentioned previously, calcein-AM was utilized to ensure labelling of biologically active intact EVs, which can be applied by spot count in the calcein channel (Fig. 4iv). Finally, to increase the accuracy of tetraspanin expression on EVs, only in-focus events were gated (Fig. 4v). Note that this gate is only applied when examining cargo, and not used for enumeration of EVs.

Using primary HBEC culture supernatant samples grown in multiple ALI inserts, samples were stained with calcein-AM and the ‘focussed EV’ gate was applied to quantify EVs in each sample (Fig. 4b). A HBEC supernatant sample without calcein staining (unstained), PBS buffer with calcein, culture medium with calcein, and a calcein-stained EV sample with 10% SDS detergent were used as controls.

### Tetraspanin profiling

#### Limit of detection

After having established that EVs can be identified by calcein-AM staining on each FC instrument, we aimed to optimise an antibody panel for tetraspanin quantification. Before staining EVs with tetraspanin antibodies, the limit of detection for potential antibody fluorophores were calculated on both the CytoFLEX S and ImageStream X MkII. The CytoFLEX nano was not tested for antibody LoD due to restricted availability of the instrument at the time of publication.

The limit of detection (LoD), in terms of antibody binding capacity (ABC), for the fluorophores APC, PE, PE-Cy5, PE-Cy7 and PE-Vio615 were quantified to assess their potential suitability for small particle characterisation. Fluorophores were selected for their relative brightness, as well as by colour to complement EV staining with FITC Calcein-AM. The LoD’s of each fluorophore were assessed on both the CytoFLEX S and ImageStream X MkII using Quantum™ Simply Cellular (QSC) Mouse and Human IgG Microspheres. Each machine’s gain and laser strengths were set to their maximum values, in accordance with established EV acquisition settings. LoDs were calculated using the manufacturer’s supplied analysis template and represent the ABC equivalent to autofluorescence of an unstained blank population of beads.

The LoDs for each fluorophore, as well as the R values, are shown in Table 1. The ImageStream X MkII generated more sensitive LoDs than the Cytoflex for all fluorophores, with the exception of PE. The Fluorophores APC and PE-Cy5 were also shown to have low LoD values for both machines, emphasising the suitability of these fluorophores on either platform, while the observed variation in PE-Vio615’s LoD suggests its suitability is machine-dependent.

**Table 1:** LoDs for fluorophore assessed on both the ImageStream X MkII and CytoFLEX S. Values generated using manufacturer’s suppliers’ analysis template.

	ImageStream X MkII		CytoFLEX S		
	LoD (ABC)	*R* Coefficient	LoD (ABC)	*R* Coefficient	
PE-Cy5	24	0.9995	87	0.9830	
APC	46	0.9673	88	0.9586	
PE-Vio615	638	0.9991	3075	0.9960	
PE	954	0.9985	241	0.9957	
PE-Cy7	1183	0.9973	2800	0.9927	

#### Tetraspanin staining

Once the limit of detection for each instrument was determined, antibodies were selected based on the LoD results. Specifically, PE-Cy7 was avoided due to its high LoD threshold, thus reduced sensitivity for detection. Subsequently, anti-CD9 APC, anti-CD63 PE, and anti-CD81 PE-vio615 tetraspanin antibodies were chosen based on their LoD values, suitability for use on the ImageStream X MkII, and their commercial availability. Tetraspanin staining on the CytoFLEX S was not carried out due to the inability to measure tetraspanins on EVs smaller than 126 nm, which is the focus of this HBEC research. Thus, the following experiments utilised the ImageStream X MkII only.

Due to the sensitivity of the 642 nm laser on the ImageStream X MkII, the max strength was reduced to 2mW to prevent the machine crashing due to increased ‘noise’. This increased the LoD to 259 ABC, though this was still more sensitive than the other channels (See supplementary Table 1 for LoD and R Coefficients when red laser intensity is reduced).

**Fig. 5:**
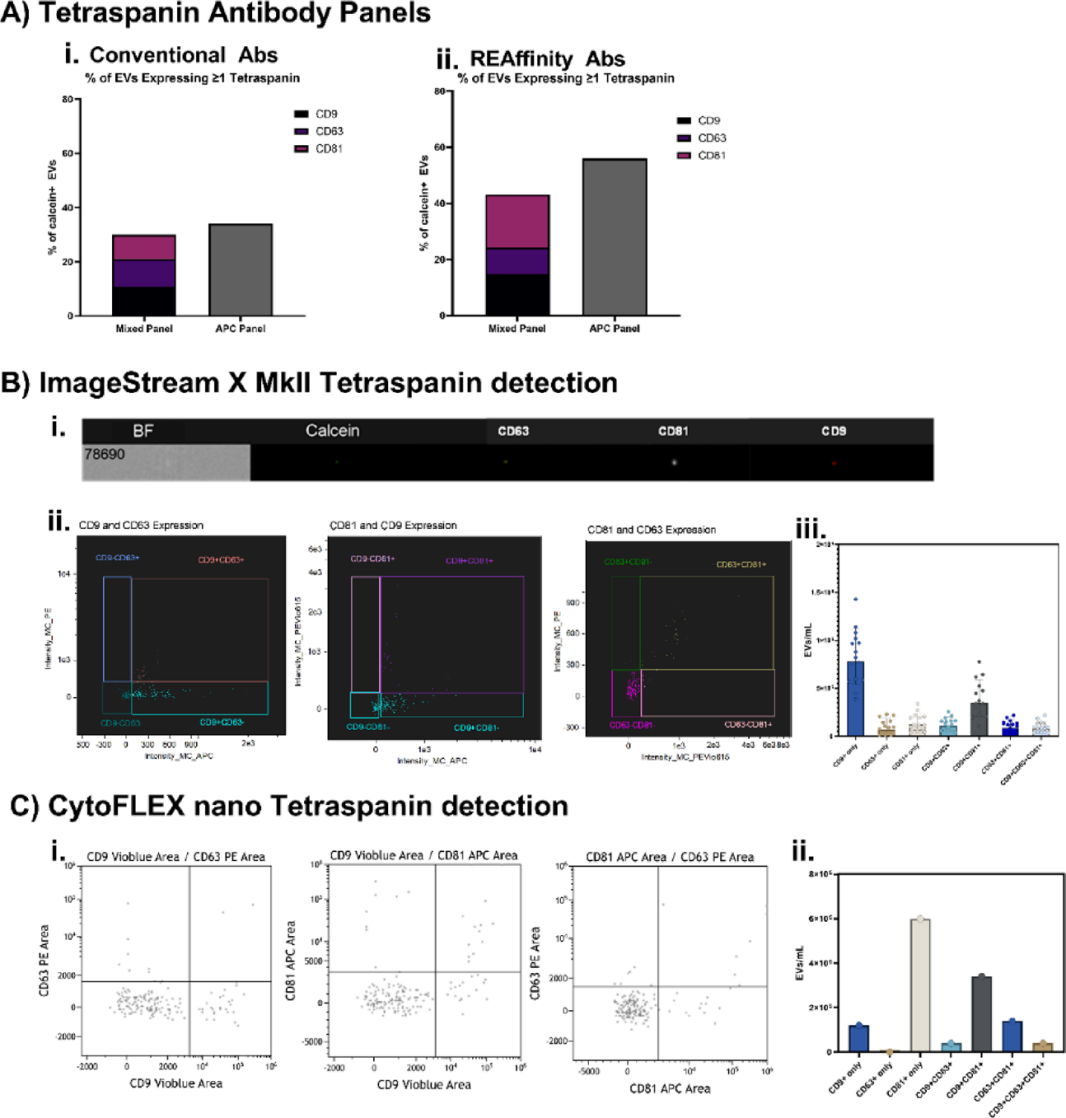
Tetraspanin Profiling. **(A)** A comparison between (i) conventional antibodies and (ii) REAfinity antibodies, as both a mixed fluorophore panel (CD9 APC, CD63 PE, and CD81 PE-Vio615) and a pan-APC panel (*N* = 1). **(B)** (i) ImageStream X MkII imaging of a single EV showing the EV brightfield image (BF), and calcein, CD63 PE, CD81 PE-Vio 615, and CD9 APC fluorescence. (ii) Bi-variate dot plots of tetraspanins markers. (iii) The number of calcein^+^ EVs expressing tetraspanin combinations, using human primary epithelial cells, on the ImageStream X MkII, with 95% confidence intervals (*N* = 15). (**C**) (i) Bi-variate dot plots of tetraspanins markers on the CytoFLEX nano. (ii) The number of calcein^+^ EVs expressing tetraspanin combinations, using human primary epithelial cells, on the CytoFLEX nano (*N* = 1).

Before characterising tetraspanins, antibodies from two companies were assessed to decipher their suitability for EV staining. Firstly, conventional mouse monoclonal antibodies and REAfinity antibodies (recombinant human antibodies with high purity and a mutated Fc region to eliminate background), were compared for their ability to detect tetraspanins (Fig. 5A). EVs were stained with calcein-AM, with either a mixed fluorophore panel (APC, PE, and PE-vio615) or a pan-APC panel. Despite all antibodies being used at the same concentration of 2.5 µg/mL, the REAfinity antibodies resulted in notably higher tetraspanin detection (mixed = 43.24% gated of EVs, pan = 56.05% gated of EVs) compared to the conventional antibodies (mixed = 30.05% gated of EVs, pan = 34.11% gated of EVs). Furthermore, a mixed fluorophore panel resulted in less sensitive detection of tetraspanins compared to the pan-APC panel, in both conventional antibodies and REAfinity antibodies, showing a pan-APC antibody panel results in more sensitive detection of tetraspanin expression on EVs. This was a proof-of-concept experiment and so it must be noted that the results are of *n* = 1.

For this study, the mixed fluorophore panel was chosen, using the REAfinity tetraspanin antibodies. Although this provides reduced sensitivity compared to the pan-APC panel, the mixed antibody panel allows further classification of EVs based on which of the 3 tetraspanins they are expressing. Without the need for prior EV isolation, supernatants from primary bronchial epithelial cells were collected and subsequently stained with calcein-AM and the mixed REAfinity tetraspanin panel, before analysing on the ImageStream X MkII (Fig. 5Bi). Tetraspanin gates set using isotype controls were applied (Fig. 5Bii) on 15 samples of HBEC culture supernatants, and the results show a quantification of calcein + EVs expressing only a single tetraspanin, as well as EVs expressing two or three tetraspanins, using the optimised methods (Fig. 5Biii).

This method can be applied to the CytoFLEX S, but tetraspanin expression would not include EVs smaller than 126 nm, based on our sizing data. This method can also be applied to the CytoFLEX nano, but would require a different fluorophore panel to the one optimised here, due to the incompatibility of PE-Vio615 with the instrument’s available lasers. For proof of principle, the following REAfinity antibodies: CD9 Vioblue, CD63 PE, and CD81 APC were used to stain EVs in conjunction with calcein-AM, and the results show it was also possible to distinguish between EVs expressing one, two or three tetraspanins (Fig. 5C). It must be noted that these HBEC-EVs were isolated by SEC before analysis on the CytoFLEX nano, as recommended by the manufacturer.

### Technique comparison summary

Table 2 summarises the flow cytometry techniques evaluated in this study. It highlights where EV isolation is required, as well as the capacity to quantify, size and characterise EVs.

**Table 2:** Summary table comparing all FC technologies used in this study for HBEC-EV analysis.

Machine	Require EV isolation	EV quantification	Sizing sensitivity	Tetraspanin characterisation
Conventional FC (CytoFLEX S)	No	Yes- number based on a flow rate of 10 µL EVs/minute	> 80 nm but 126 nm when using VSSC to size	Yes- Up to 6 fluorophores + calcein, but only on EVs above 126 nm
Imaging FC (ImageStream X MkII)	No	Yes- number based on a flow rate of 1 µL EVs/minute	> 55 nm	Yes- Up to 6 fluorophores + calcein, on EVs larger than 55 nm
Nanoscale FC (CytoFLEX nano)	Recommended – used in this study	Yes- number based on a flow rate of 1 µL EVs/minute	> 40 nm	Yes- 3 fluorophores + calcein

## Discussion

EVs secreted by human bronchial epithelial cells (HBEC-EVs) have been demonstrated in regulating immune responses in the lung, proving to be key cell communicators in respiratory diseases^3–7^. However, there is a lack of research utilising HBEC-EVs, especially with standardised methodology for EV analysis. With several techniques currently required to accurately measure EV numbers, size and tetraspanin expression, such as NTA and western blotting, flow cytometry provides the ability to fully characterise EVs using a single technology. Flow cytometry can also provide high-throughput single-EV analysis, which can enhance research in the EV field and allow application to clinical settings. With emerging flow cytometry techniques, the evaluation of different flow cytometry methods for EV analysis is lacking. Here, we further develop methods previously published^19–26,30^ for HBEC-EV quantification, sizing, and tetraspanin characterisation and apply these methods using a range of FCs: conventional, imaging, and nanoscale. This study aims to provide clarity on what methods and instruments are best suited to study HBEC-EVs, which has not been shown previously.

In the present study, to identify HBEC-EV size distributions, multiple analytical techniques were compared: NTA (Zetaview), conventional FC (CytoFLEX S), nanoscale FC (CytoFLEX nano), and imaging FC (Imagestream X MkII). NTA has a particle concentration-dependent sensitivity profile across 20–500 nm detection range, which is considered the most commonly used for sizing EVs^31^. It relies on light scattering and Brownian motion analysis to calculate the relative EV sizes. Here we show measurement of HBEC-EV sizes of approximately 100–200 nm, which was consistent with the typical size ranges for exosomes (30–150 nm) and smaller microvesicles.

In comparison to NTA, flow cytometry uses light scattering and fluorescence for EV analysis. When sizing EVs by flow cytometry, the scattered light must exceed the triggering threshold (when triggering on SSC), which must be set to exclude any optical and electronic noise. Whilst this is easy to apply for large EVs or cells, this is difficult for smaller EVs, which produce very low scatter signals, falling within the electronic noise and so cannot be detected. Here we show that conventional flow cytometry was the least sensitive, as it was unable to distinguish between background and HBEC-EVs below 126 nm in size, using VSSC, NIST particles and Mie theory size calculations. This is in agreement with previous research^32^, which also concluded that conventional FCs are inadequate for smaller EV analysis. It is noteworthy that this sensitivity was low despite measuring SSC off the violet laser (VSSC) instead of the conventional blue laser, which is more sensitive than regular SSC as the 405 nm wavelength of the violet laser is closer to EV size compared with the 488 nm wavelength of the blue laser^33^. Both small and large HBEC-EVs are relevant to respiratory diseases, however, small EVs are most researched due to their potential in delivery and therapeutic applications^34^, but it is important to note that this instrument may still be viable if aiming to characterise larger HBEC-EVs.

The CytoFLEX nano utilizes the same Mie theory and FCMPASS calculations based on reference beads as the CytoFLEX S, however, here we show that the sensitivity of the nano flow cytometer improves the resolution of EVs down to 40 nm in size. This highlights its enhanced sensitivity for sub-200 nm particle analysis due to its refined optical detection limits.

When sizing HBEC-EVs using the ImageStream X MkII, due to a lack of software availability to calculate size estimation using Mie Theory, an alternative approach using fluorescent lipid dye incorporation was utilised. Using a liposome size standard correlated with surface area-to-fluorescence of a membrane dye, vFRed, the ImageStream X MkII estimated sizing of HBEC-EVs to be as small as 55 nm. Sizing EVs using fluorescence rather than VSSC can be more sensitive and in practise is often easier to achieve^32,35^. The signal is often much brighter relative to background noise than scatter signals for very small particles, enabling better discrimination and detection. Hence, it may be beneficial to adopt fluorescence-based triggering for EV sizing rather than using VSSC. Overall, the sizing results suggest imaging and nanoscale FCs are more appropriate for small EV analysis compared to current conventional FCs.

Importantly, conventional and imaging flow cytometry sizing methods were developed without the need for prior isolation of EVs, removing any selection bias of EVs and reducing time to analysis, which is specifically advantageous in a clinical setting. However, nanoscale cytometers recommend prior EV isolation before analysis. Thus, the choice of instrument used heavily depends on the research question, source of EVs and their expected sizes. It must be highlighted that due to differences in sample preparations for the CytoFLEX nano compared to the CytoFLEX S and ImageStream X MkII, the differences in EV sizes are not directly comparable. Accordingly, the data should be interpreted as illustrating instrument-specific performance and methodological demands, rather than absolute differences in EV size.

In terms of EV detection and enumeration by flow cytometry, calcein-AM dye was used in this study as it allows for discrimination between biologically active intact EVs and debris, due to its hydrolysis into a fluorescent analogue when cleaved by an intravesicular esterase. In addition, calcein-AM has successfully been used to stain EVs previously^14,24,36,37^. We provide optimised methods for detecting calcein-positive EVs using conventional, nanoscale, and imaging FCs. The calcein-AM dye was tested at 8 different concentrations, with a concentration of 10 µM determined optimal based on the best stain index. This can then be used in conjunction with fluorescent tetraspanin antibodies to enumerate EVs as well as phenotype marker expression. One limitation of detecting EVs with calcein-AM is the assumption that all EVs contain esterase, which is incorrect^38^, resulting in the exclusion of EV subpopulations in the analysis. Future work could incorporate tandem staining for EV enumeration, using calcein-AM alongside a lipid-membrane dye, where double-positive staining would then indicate all intact EVs within the sample.

The characterisation and profiling of tetraspanin expression on the surface of EVs is standardised practice within the field of EV research^1,22^. While tetraspanin expression on EVs is not fully understood, they represent an important biomarker with highly varied expression that can be an indicator of both an EV’s cellular origin, as well as its potential function^1,39–41^. While there are a variety of ways to profile the expression of these markers, such as Western Blot analysis, flow cytometry provides an approach to quantify this expression. Indeed, flow cytometry does not just quantify a marker’s presence, but it also measures the individual and combined expressions of tetraspanin markers on single EVs, as well as across a whole population. However, the transition from flow cytometry’s original purpose of characterising cellular populations to characterising EV populations requires a variety of considerations to be effectively implemented at the small particle scale. A key consideration is the sensitivity of the cytometer. At the cellular level, the literature suggests that tetraspanins are expressed at about 30,000–100,000 copies per cell^42^ ultimately providing adequate binding sites for enough antibodies to create a detectable signal for the cytometer. While the heterogeneous and varied nature of tetraspanin expression on EVs is established in the literature^22,39,41^, how this relates to copy numbers on an EV is unknown. Consequently, to avoid potential false negative profiling as a result of low copy number, the limit of detection (LoD) of the assay should be measured. Thus, in this study, the LoD of different fluorophores were measured on the CytoFLEX S and ImageStreamX MkII in terms of antibody binding capacity (ABC). Understanding LoDs in the context of a minimum number of bound antibodies required to trigger a detectable event or ABC provides a platform for a more practical assessment of a fluorophore’s suitability for small particle tetraspanin analysis and characterisation, as it can be related to potential copy numbers on EVs. Here, we highlight not just the importance of traditional fluorophore selection based on colour separation, but also on the cytometer being used, as LoDs vary on each FC. For example, on the CytoFLEX S, the LoDs ranged from 87 to 3075 depending on the fluorophore, whereas on the ImageStream X MkII it ranged from 24 to 1183. While an LoD of 3075 would be adequate for profiling cells with 30,000 or more copies, how this relates to an EVs is less clear and should be taken into consideration. Consequently, identifying and using fluorophores with low LoDs, such as APC and PE-Cy5, would be preferable. In addition, selection of a panel of fluorophores with not just low, but also similar LoDs, would be preferable to remove any bias in fluorophore detection which may impact tetraspanin quantification when measuring multiple tetraspanins simultaneously. It is worth noting that the LoDs presented here are calculated from the autofluorescence measured from the blank bead population provided, which are larger than EVs, and could mean that LoD values quoted here are an overestimate. Consequently, LoDs could be measured using the autofluorescence of unstained samples to provide more accurate LoDs for EV analysis.

Tetraspanin antibodies were subsequently chosen based on low LoD values and commercial availability, using anti-CD9 APC, anti-CD63 PE, and anti-CD81 PE-Vio615. Antibodies were used at 2.5 µg/mL, based on previous literature using a range of 1.25–6.6^19,32^. Due to focusing on smaller EVs, tetraspanin-stained EVs were not analysed on the CytoFLEX S, as we showed no detection of EVs below 126 nm. Thus, tetraspanins on bronchial epithelial EVs were analysed on the CytoFLEX nano and ImageStream X MkII. We showed detection of all three tetraspanins by both FCs. Thus, using these optimised methods, we were able to quantify EVs, phenotype for one, two or three tetraspanins, and infer their minimum ABC value based on the LoD data.

While the novelty of this study lies in its focus on HBEC-derived EVs, the flow cytometry approaches and methodological considerations described are broadly applicable across the EV field. The principles governing EV detection, sizing, and phenotyping, particularly instrument sensitivity, scatter versus fluorescence-based triggering, fluorophore limit of detection, and the use of appropriate EV-specific controls, are relevant to EVs derived from diverse biological systems. In more clinically relevant settings, where EV populations are heterogeneous and originate from multiple cell types, these considerations become even more critical due to increased background and particle complexity. Imaging and nanoscale flow cytometry platforms are especially well suited to such samples, including plasma, enabling single-EV resolution and multiparametric phenotyping without reliance on bulk isolation methods that may bias EV subpopulations^19^.

Overall, we show conventional FCs, such as the CytoFLEX S, with higher sample flow rate, are not capable of detecting HBEC-derived small EVs using violet side scatter thresholding (diameters < 100 nm). Imaging (ImageStreamX MkII) and nanoscale (CytoFlex nano) FCs provide higher sensitivity, but with slower sample flow rates. Importantly, imaging and conventional cytometers allow the analysis of EVs without prior need for isolation, enabling analysis of a whole sample, without losing EVs through methods such as SEC. We also highlight the need for thorough considerations before EV analysis, such as LoD measurement of fluorophores to create a robust phenotyping panel. In conclusion, we provide strategies and considerations for the enumeration, sizing, and phenotyping of HBEC-EVs using a single flow cytometry technology, which is high-throughput and requires limited sample preparation.

## Supplementary Information

Below is the link to the electronic supplementary material.

Supplementary Material 1

## Data Availability

All data required to evaluate the conclusions in the paper are present in the manuscript or its supplementary material. Further information is available from the corresponding authors on reasonable request.
