# Supplementary material for: Bronchial epithelial cell-derived extracellular vesicle analysis using conventional, imaging, and nanoscale flow cytometry technologies
Source: Sci Rep. 2026 Feb 26;16:11162. doi: 10.1038/s41598-026-41848-x (PMC13046774; doi:10.1038/s41598-026-41848-x)
Supplement: Supplementary file 1 — Supplementary Material 1 [file 41598_2026_41848_MOESM1_ESM.docx]

# **Supplementary Information**

**Supplementary Methods**

***FC Configuration and Gain Settings***

CytoFLEX S: The V405 filter was manually swapped with the V450 filter on the violet laser detector pod and the detector configuration settings on the machine, using CytExpert 2.5 software, were changed accordingly from default to violet side scatter (VSSC) configuration. Sample flow rate was set to ‘slow’, equivalent to 10 uL/min. Primary trigger was VSSC, at 40k threshold.

CytoFLEX nano: The primary tigger was VSSC1 at a threshold of 265. Gains for V447 (542), B531 (994), Y595 (827), and R670 (438).

ImageStream X Mk II: Both camera 1 and 2’s sensitivities were set to 32 for all channels and both camera’s gains were 1. The ImageStream X MkII was equipped with a 405nm (120mW) 488 nm (400mW), 561 nm (200 mW) and 785 nm (100mW). The 642 nm was used at a 150 mW for sizing and LoD analysis, but used at a reduced power of 2 mW for tetraspanin analysis to reduce machine errors when using APC-conjugated antibodies. Fluidics settings set to “low speed/high sensitivity” resulting in a flow speed of 44 mm/sec, and a sample core size of 7 µm.

**Table S1.** LoD values when reducing max strength of red (642nm) laser.

|  | LoD (ABC) | R Coefficient |
| --- | --- | --- |
| APC 2mW | 259 | 0.9843 |
| APC 50mW | 49 | 0.9774 |
| APC 100mW | 47 | 0.9718 |
| APC 150mW | 46 | 0.9673 |
